# Identification and characterisation of the ecdysone biosynthetic genes *neverland*, *disembodied* and *shade* in the salmon louse *Lepeophtheirus salmonis* (Copepoda, Caligidae)

**DOI:** 10.1371/journal.pone.0191995

**Published:** 2018-02-05

**Authors:** Liv Sandlund, Heidi Kongshaug, Tor Einar Horsberg, Rune Male, Frank Nilsen, Sussie Dalvin

**Affiliations:** 1 Sea Lice Research Centre, Department of Biological sciences, University of Bergen, Bergen, Norway; 2 Sea Lice Research Centre, Institute of Marine Research, Bergen, Norway; 3 Sea Lice Research Centre, Norwegian University of Life Sciences, Oslo, Norway; Institute of Zoology Chinese Academy of Sciences, CHINA

## Abstract

The salmon louse is a marine ectoparasitic copepod on salmonid fishes. Its lifecycle consists of eight developmental stages, each separated by a molt. In crustaceans and insects, molting and reproduction is controlled by circulating steroid hormones such as 20-hydroxyecdysone. Steroid hormones are synthesized from cholesterol through catalytic reactions involving a 7,8-dehydrogenase Neverland and several cytochrome P450 genes collectively called the Halloween genes. In this study, we have isolated and identified orthologs of *neverland*, *disembodied* and *shade* in the salmon louse (*Lepeophtheirus salmonis*) genome. Tissue-specific expression analysis show that the genes are expressed in intestine and reproductive tissue. In addition, levels of the steroid hormones ecdysone, 20-hydroxyecdysone and ponasterone A were measured during the reproductive stage of adult females and in early life stages.

## Introduction

The salmon louse, *Lepeophtheirus salmonis*, is an ectoparasitic copepod (*Copepoda*, *Caligida*) infecting salmonid fishes. The parasite feeds on the mucosa, skin and blood of the host creating open wounds that increase the chance of secondary infections and mortality. The lice cause a serious threat to farming of Atlantic salmon in Norway, UK, USA and Canada and are estimated to contribute to an annual loss of more than 500 million USD in Norway alone http://nofima.no/nyhet/2015/08/kostnadsdrivere-i-oppdrett/.

The life cycle consists of eight developmental stages: nauplius I and II, copepodid, chalimus I and II, pre-adult and adult stage, each separated by a molt [1–3]. Adult females produce batches of oocytes that are generated in coiled tubular structures that form the ovaries in the anterior part of the animal and are transported to the genital segment through the oviduct [4]. The eggs mature inside the genital segment where lipids and vitellogenins are incorporated [5, 6]. The eggs are fertilized and extruded from the female in long strings containing several hundred eggs. Both oocyte maturation in the genital segment and embryogenesis take approximately 10 days at 10°C in *L*. *salmonis* [5].

In arthropods, embryogenesis and molting are regulated by ecdysteroid hormones, a group of cholesterol derived poly-hydroxylated ketosteroids that in addition to molting, are responsible for regulating essential endocrine regulated processes such as embryogenesis, growth and reproduction. Ecdysteroidogenesis takes place in special molting glands known as the prothoracic glands (PG) and Y-organ (YO) in insects and decapod crustaceans, respectively. In contrast to the decapods, molting glands has not been identified in crustaceans like the salmon louse but it has been suggested that these species possibly synthesise ecdysteroids in the epidermis [7–9]. Regulation of ecdysteroid synthesis is complex and is controlled by peptide hormones. While the prothoracicotropic hormone (PTTH) stimulates ecdysteroid synthesis in insects, the Y-organ is under inhibitory control of the molt-inhibiting hormone (MIH) [10]. However, the synthesis pathway of ecdysteroids is similar in insects and crustaceans suggesting that the pathway was present in their common ancestor [7, 11–13].

Arthropods are unable to synthesise cholesterol *de novo* and uptake of exogenous cholesterol through the diet is necessary [14–16]. In *Drosophila melanogaster* (*D*. *melanogaster*), a number of enzymes known to be involved in the synthesis has been described and orthologs of these have in recent years been identified in crustaceans [17–19]. The enzymes include Neverland (Nvd) and the cytochrome P450 mono-oxygenases (CYP450) Spook (Spo; CYP307A1), Phantom (Phm: CYP306A1), Disembodied (Dib: CYP302A1), Shadow (Sad: CYP315A1) and Shade (Shd: CYP314A1) that are collectively called the Halloween genes. The initial reaction in the conversion of dietary cholesterol to 7-dehydrocholesterol (7dC) is performed by the 7,8-dehydrogenase Nvd [20, 21]. Loss of Nvd function in the prothoracic gland (PG) of *D*. *melanogaster* and *Bombyx mori* results in an arrest of both growth and molting (20). 7dC is converted to another intermediate 5β-diketol by enzymes coded from *non-molting glossy* (*nm-g*)/*shroud* (*sro*), *spook* (*spo*) and *spookier* (*spok*) in several steps called “the black box” [17, 22–24]. 5β-diketol is, through sequential hydroxylations, modified into the secreted steroid, which in crustaceans include ecdysone (E), 3-dehydroecdysone (3DE), 25-deoxyecdysone (25DE) and ponasterone A (PonA, 25-Deoxyecdysterone) [10, 25–28]. These products are released into the hemolymph and transported to peripheral tissues where they are converted into the biologically active metabolites 20-hydroxyecdysone (20HE) or ponasterone A (PonA) by Shd [7, 10, 29, 30]. 20HE acts by binding to a heterodimer complex consisting of the two nuclear transcription factors, the ecdysone receptor (LsEcR) and retinoid X receptor (LsRXR). In *L*. *salmonis*, *Lsecr* and *Lsrxr* are expressed in many tissues including the intestine, sub-cuticular tissue, ovaries and oocytes [31–33]. The sub-cuticular tissue in *L*. *salmonis* has been demonstrated to have functions similar to the liver [34] and is the production site for yolk proteins [5, 35]. Synthesis of yolk proteins has been associated with an increase in 20HE level in the isopod *Porcellio dilatatus* [36].

In crustaceans and insects, the 20HE level in the hemolymph peaks right before molting followed by an immediate decline in concentration after ecdysis [37, 38]. Hormone levels appears to directly mimic the expression level of their synthesizing enzymes as has been observed in *B*. *mori* and *Manduca sexta* during development.

The aims of the present study were to identify and assess the expression pattern of *neverland* and the Halloween genes *disembodied* and *shade* and investigate their significance in the biosynthesis of ecdysteroids in the salmon louse. These genes were cloned and sequenced and ontogenetic expression analysis using RT-qPCR as well as transcript detection using in situ were performed. In addition, we measured the level of the ecdysteroid hormones E, 20HE and PonA in different life stages of the lice.

## Materials and methods

### Salmon lice

A laboratory strain of the Atlantic salmon louse *Lepeophtheirus salmonis salmonis* [39] was maintained and cultivated on Atlantic salmon (*Salmo salar*) [40]. Both lice and fish were kept in seawater with salinity of 34.5 ppt and with temperature at 10 ± 0.2°C. Eggs were hatched and cultivated to copepodid stage in flow-through incubators [40]. After infection of salmon the lice were kept on the fish until they reached the desired developmental stage. Prior to sampling of lice, the fish was either killed with a blow to the head or anaesthetized in a mixture of methomidate (5 mg/l) and benzocaine (60 mg/l) to minimize suffering; thereafter lice were removed with forceps. All experimental procedures were in accordance with the Norwegian legislation for animal welfare. All experiments were approved by The Animal Ethics Committee by The Norwegian Food Safety Authorit (Permit Number: 8589).

### Cloning, sequence analysis and alignment

Candidate genes from the cytochrome P450 enzymes CYP314a1/*shade (shd)* and CYP302a1/*disembodied* (*dib*) and the 7,8 dehydrogenase *neverland* (*nvd*) known to be involved in the synthesis of ecdysteroids were identified in the salmon louse genome (www.Licebase.org) by homology to known sequences from insects and crustaceans. Sequences with the lowest e-value were chosen. Gene specific 5`and 3`RACE primers were designed (Table 1) and RACE was performed using the SMARTer^™^RACE cDNA Amplification kit (Clontech, Mountain view, CA, USA) according to manufacturer’s recommendations (Sigma-Aldrich, St. Louis, MO, USA). Sub-cloning was performed using a pCR^®^4-TOPO^®^ vector system (Invitrogen, Carlsbad, CA, USA) that were transformed into *Escherichia coli* TOP10 cells. Clones were verified by PCR with M13_f and M13_r primers (Table 1), grown overnight and purified using a Miniprep Nucleospin^®^ Plasmid Purification Kit (Macherey-Nagel, Duren, Germany). Plasmids were sequenced using a BigDye^®^ Terminator v3.1 Cycle sequencing kit (Applied Biosystems^®^, Foster City, CA, USA) and analyzed in MacVector (MacVector Inc., NC, USA). Sequencing was performed by the Sequencing Facility at the Molecular Biological Institute, University of Bergen.

**Table 1:** Primer sequences and SYBR^®^ Green assays used in this study.

Primer name^b^	Sequence (5`-3`)	Method
*Lsnvd_5´RACE*	GTCACAGGTCTCCACAAGGGTCTTTCAG	RACE
*Lsnvd_3´RACE*	CTGAAAGACCCTTGTGGAGACCTGTGAC	RACE
*Lsdib_5´RACE*	TTCCACAGGGGAGGTCCATTGTCCG	RACE
*Lsdib_3´RACE*	CTGTGCCGAAAGGGACTGTTCTTGTGAG	RACE
*Lsshd_5´RACE*	GCGAGCAGTGTCCAAGGCAATAGTGTCG	RACE
*Lsshd_3´RACE*	CCTTGCCGACACTATTGCCTTGGACACT	RACE
*Lsshd_3´RACE_nested*	TCACAGCAGGAGTAGACACCATAGG	RACE
M13_f	GTAAAACGACGGCCAG	Topo cloning
M13_r	CAGGAAACAGCTATGAC	Topo cloning
*Lsnvd_P1_F*	GACCCTTGTGGAGACCTGTG	*In situ*
*Lsnvd_P1_R*	TTGGCAATGTGGGGATTGGT	*In situ*
*Lsdib_P1_F*	CCTCCCCTGTGGAAGGTTTTT	*In situ*
*Lsdib_P1_R*	GAACAGTCCCTTTCGGCACA	*In situ*
*Lsshd_P1_F*	ACCGTCATTTTCGCCCTTCT	*In situ*
*Lsshd_P1_R*	AGGCTCTTGTTGTGTGCGTA	*In situ*
*Lsef1α_F_ SYBR*^*®*^ ^a^	CATCGCCTGCAAGTTTAACCAAATT	RT-qPCR
*Lsef1α_R_ SYBR*^*®*^ ^a^	CCGGCATCACCAGACTTGA	RT-qPCR
*Lsnvd F_SYBR*^*®*^ ^a^	AACCAATCCCCACATTGCCA	RT-qPCR
*Lsnvd R_SYBR*^*®*^ ^a^	GGCCAGAAGCGATTTGTGTA	RT-qPCR
*Lsdib_F_ SYBR*^*®*^ ^a^	ACCGTCATTTTCGCCCTTCT	RT-qPCR
*Lsdib_R_ SYBR*^*®*^ ^a^	GCCTGGAGGAAGTGAGTGTC	RT-qPCR
*Lsshd_F_ SYBR*^*®*^ ^a^	GCTATGGGCCTGTAGTGAGG	RT-qPCR
*Lsshd_R_ SYBR*^*®*^ ^a^	AACATCCGCCTCATTCGGAG	RT-qPCR
*Lsecr_F_ SYBR*^*®*^ ^a^	TCGCCCAACTCACGATTCAG	RT-qPCR
*Lsecr_R_ SYBR*^*®*^ ^a^	GGGGAGTAAGGATGGGGTTC	RT-qPCR
*Lsrxr_F_ SYBR*^*®*^ ^a^	CCTAGTTGAACTCATCGCCAAAATG	RT-qPCR
*Lsrxr_R_ SYBR*^*®*^ ^a^	TGAAGAGTATGATGGCTCGTAGACA	RT-qPCR

^a^ SYBR^*®*^ Green assays were provided by Applied Biosystems, Branchurg, NJ, USA.

^b^ All general primers were purchased from Sigma-Aldrich, St Louis, MO, USA.

RACE, rapid amplification of cDNA ends; TOPO, DNA topoisomerase I; RTq-PCR, real-time quantitative PCR

### Collection of salmon lice

#### Lice for ontogenetic analysis

For each analysis, three or five parallels of n = x individuals for each developmental stage of the *L*. *salmonis* were collected; nauplius I/II (naup. I/II) and free-living copepodids (Free. Cop.) (n = 5 x (≈ 150), parasitic copepodids (Par. Cop.) (n = 3 x 10), chalimus I (Chal. I) and chalimus II (Chal. II) (n = 5 x 10), pre-adult male I/II (Pre-A. M. I/II), pre-adult female I/II (Pre-A. F. I/II), adult male (Adult M.) and immature adult female lice (Adult F.) (n = 5 x 1) and stored on RNAlater^™^ (Ambion inc., Foster City, CA, USA).

#### Lice for LC/MS/MS analysis

From the moment the egg strings are fertilized, it takes approximately 10 days at 10°C for them to mature and hatch. In order to determine the presence of ecdysteroid and the ecdysteroid levels during the maturation of the egg strings, adult female lice were sampled and their corresponding egg strings were transferred to individual incubators with flowing seawater at 10°C. The time of hatching were noted for each egg string were noted and used to determine the exact point of the egg maturation cycle for each female. Five biological samples for each time point (10 days) and all together 50 female lice were collected and frozen dry at– 80°C. In addition, we collected nauplius II (n = 3 x 200), free-swimming copepodids (n = 3 x 200) and parasitic copepodids (n = 3 x 30) two days post infection (d.p.i.), in order to determine the ecdysteroid content during larval stages.

### Detection of transcript using in situ hybridisation

Salmon louse were fixed in 4% paraformaldehyde in phosphate buffered saline (PBS) for 24 hours and transferred to 70% ethanol at 4°C for at least 24 hours before paraffin embedding. *In situ* hybridization was carried out according to [41], with the same modifications as described in [42]. Digoxigenin (DIG)-labelled anti-sense and sense RNA probes (*Lsnvd*: 261 bp, *Lsshd*: 327 bp, *Lsdib*: 475 bp) were generated from PCR templates (Table 1) with T7 promoters using DIG RNA labelling kit (Roche Diagnostics GMbH, Mannheim Germany). 25 ng/μl probe was used in the hybridisation mix for detection of *Lsshd*, *Lsdib* and *Lsnvd*, respectively. Chromogenesis was carried out using nitroblue tetrazolium (NBT) (Roche Diagnostics GMbH) and 5-bromo-4-chloro-3-indolyl phosphate (BCIP) (Roche Diagnostics GMbH). Sense RNA was used as a negative control.

### RNA extraction and cDNA synthesis

Total RNA from animals collected from RNAi experiments and for ontogenetic analysis was isolated using TRI Reagent^®^ (Sigma-Aldrich) as previously described by [42], according to the manufacturers protocol. Animals were homogenised in Tri Reagent^®^ and added chloroform. After extraction of the water phase, RNA from nauplius and copepodid samples were extracted using RNeasy micro kit (Qiagen, Hilden, Germany) according to the manufacturer`s protocol. RNA was treated with DNase I (Amplification Grade, Invitrogen). Samples were stored at -80°C. cDNA synthesis was achieved using AffinityScript qPCR cDNA synthesis Kit (Agilent Technologies, Santa Clara, CA, USA) according to the protocol. cDNA was diluted 5 or 10 times and stored at -20°C.

### Real time quantitative PCR (RT-qPCR)

Real-time quantitative PCR (RT-qPCR) using SYBR^®^ green assays (Table 1) were applied to detect total expression of genes from dsRNA treated lice harvested from the RNAi experiments as well as genes submitted to ontogenetic analysis. Primers were designed using MacVector (MacVector inc.). Primers were designed to localise the respective gene independently of their specific dsRNA fragments. Eight dilutions (from 100–0.78 ng/ul) of RNA from *L*. *salmonis* were used as template to generate a dilution curve and verify the efficiency (1.87–2.1) for each assay. Reaction specificity was confirmed by a single peak in the dissociation curve was present. All assays were run in parallel series together with the reference gene *eEF1α* [43]. One reaction without reverse transcriptase was run to exclude contamination of genomic DNA. All samples were added 2 ng/μl cDNA containing, 2X SYBR^®^ Select Master Mix with ROX (Applied Biosystems^®^) and 10 μM of each primer to the total reaction volume of 10 μl. Thermal cycling and quantification were performed using Applied Biosystems 7500 Real-Time PCR System under the following conditions: enzyme activation step; 95°C 15 min, followed by 40 cycles of denaturation of 95°C for 15 sec and extension; 50–60°C for 1 min (dependent on the gene). All samples were normalised to *eEF1α* by the 2^-ΔΔCt^ approach and relative expression was calculated using controls from each experiment as standard.

### Chemicals for LC/MS/MS analysis

Ecdysone (E), 20-hydroxyecdysone (20HE) and ponasterone A (PonA) were purchased from Sigma-Aldrich. All stock solutions were made in the laboratory.

### Ecdysteroid extraction

Using a single clean scalpel, animals were divided into the cephalothorax (CT) and abdomen/genital (Ab/G) segment,1 ml acetonitrile were added and homogenized using a 5 mm stainless steel beads (Qiagen) in a Tissuelyser LT (Qiagen) for 3 x 2 min with 1 min cooling on ice between each homogenization step followed by 10 min of vortexing. The homogenate was centrifuged at 16500G for 5 min at 4°C and the supernatant was transferred to a 15 ml polypropylene (PP) tube and dried under N_2_ (12–15 psi) at 35°C using a TurboVap LV evaporator (Zymark, Bay Street, Midland, ON, Canada). Extracts were re-dissolved in 50 μl MeOH/H2O (50/50 v/v), vortexed and transferred to High Performance Liquid Chromatography (HPLC) vials and subjected to LC/MS/MS analysis. Five biological parallels were subjected to analysis for each life cycle stage analysed.

### LC/MS/MS analysis

Samples were analysed in an LC/MS/MS system consisting of a PerkinElmer series 200 HPLC system (Shelton, CT) and an API4000 triple quadruple mass spectrometer (AB SCIEX; Foster City, CA) containing an electrospray ionization source. HPLC separation was performed using a Discovery^®^ C18 HPLC Column (15 cm x 2.1 mm, 5 μm; Supelco, Sigma-Aldrich), 300 μl/min flow rate at room temp. Analytes were separated by methanol and 0.1% acetic acid under the gradient conditions presented in Table 2. Injection volume of sample was 30 μl. MS/MS analysis was performed under the following conditions: Curtain gas (CUR), 30 L/min; Collision gas (CAD), 4.0 L/min; Ion Source Gas (GS1), 20 V; Ion Source Gas (GS2), 70 V; Ion spray voltage (IS) 5000; Temperature (TEM) 400°C; Declustering potential (DP), 95.0 V; Entrance potential (EP), 9 V; Collision energy (CE), 20.0 V; Collision cell exit potential (CXP), 11.0 V. Selected reaction monitoring (SRM) was executed using the transitions specific for E, 20-E and PonA (Table 3). Obtained results were analysed using Analyst^®^ 1.6.1 Software (AB SCIEX). Concentrations of the compounds in each sample were estimated using the peak areas of the SRM chromatogram on the basis of a calibration curve for each component, constructed using a standard. For the 20HE analyte, two fraction ions were detected due to an unspecific interfering retention top. A sample of the analytes (1ng/mL) and a diluent sample (MeOH:H_2_O) was run every ten samples to track any degradation of analytes or contamination during the run.

**Table 2:** Gradient for analysis of selected ecdysteroids of this study.

Time (min)	Flow (μL/min)	Methanol %	0.1% Acetic acid
0	300	80	20
25	300	10	90
28	300	10	90
30	300	80	20
50	300	80	20

**Table 3:** SRM conditions used in tandem mass spectrometry analysis.

Ecdysteroids	Retention time (min)	SRM transition (m/z)	CE (V)
E	19.7	465/429	20
20HE	18	481/445	20
PonA	24.7	465/447	30

SRM: selected reaction monitoring, CE: collision energy, E: ecdysone, 20HE: 20-hydroxyecdysone, PonA: ponasterone A.

The evaluation of the range of linearity and calibration curves were established by injecting standard solutions of the three ecdysteroids (E, 20HE and PonA). Standard solutions prepared in methanol and in salmon louse matrix at the concentrations 0.02, 0.1, 0.5, 2.0, 4.0, 5.0 and 8.0 ng g^-1^ were run in triplicates. Five replicates of the 5.0 ng g^-1^ from the two standard curves were run in order to determine the accuracy and precision of the method. Limit of detection (LOD) and limit of quantification (LOQ) were determined in the SRM mode analysis from the lowest standard concentration injected giving a signal-to-noise (S/N) ratio of three and nine, respectively.

### Statistical analysis and bioinformatics

One-way ANOVA tests were used to determine differences in steroid levels between days in adult females and in larval stages. A significance level of α = 0.05 was used. In addition to *in situ* hybridisation, RNA-sequencing data obtained from www.Licebase.org was used to analyse the tissue expression pattern of the identified genes.

## Results

### Identification and sequencing of *neverland* (*nvd*) and the two Halloween genes *disembodied* (*dib*) and *shade* (*shd*)

**Fig 1:**
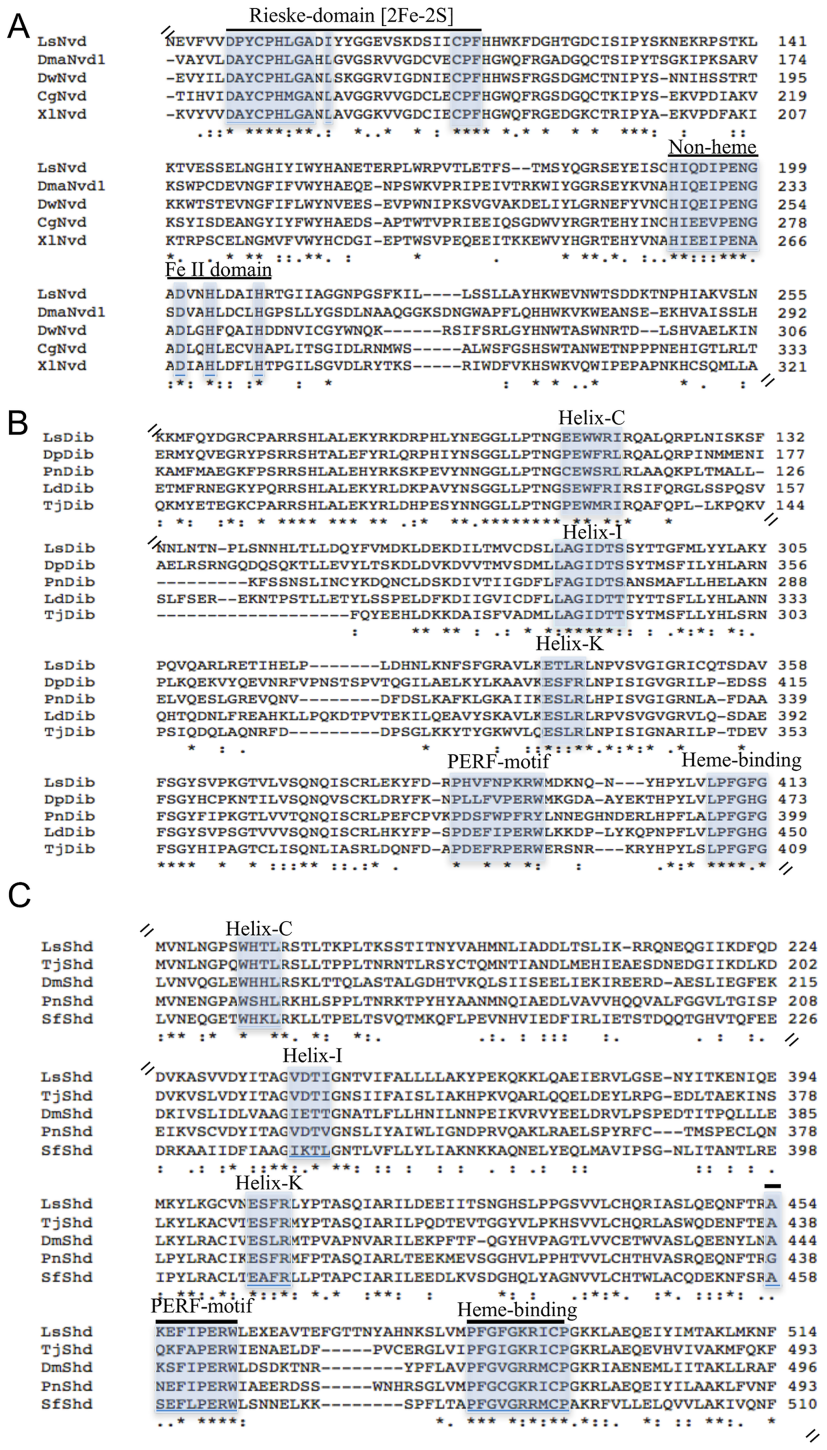
Alignment of deduced amino acid (aa) sequences of *Lepeophtheirus salmonis* 7,8-dehydrodenase/Neverland (*Ls*Nvd) (A), disembodied (*Ls*Dib) (B), and shade (LsShd) (C) with other species. A) The conserved Rieske motif and the non-heme domain are market off in the alignment. The sequences used in the alignments were as follows; *L*. *salmonis* Neverland (*Ls*Nvd; MF 598470), *Daphnia magna* Neverland subtype 1 (*Dma*Nvd1; BAQ02388.1), *Daphnia wassermani* Neverland (*Dw*Nvd; AFD97360.1), *Crassostrea gigas* Neverland (*Cg*Nvd; XP_011445555.1), *Xenopus laevis* Neverland (*Xl*Nvd; BAK39959.1). B) Conserved P450 motifs are shown. The sequences used in the alignments were as follows; *L*. *salmonis* disembodied (Lsdib; ACO13011.1), *Daphnia pulex* Disembodied (*Dp*Dib; EFX63066.1), *Paracyclopina nana* CYP 302A1 (PnDib; AKH03533.1), *Leptinotarsa decemlineata* CYP 302A1 (*Ld*Dib; AGT57842.1), *Tigriopus japonicus* CYP 302A1 (*Tj*Dib; AIL94171.1). C) Conserved P450 motifs are shown. The sequences used in the alignments were as follows; *L*. *salmonis* Shade (*Ls*Shd; MF598469), *Tigriopus japonicus* Shade (*Tj*Shd; AIL94172.1), *Daphnia magna* Shade (*Dm*Shd; BAF35770.1), *Paracyclopina nana* Shade (*Pa*Shd; AKH03534.1) and *Sogatella furcifera* Shade (*Sf*Shd; AGI92296.1).

To verify the sequences of the selected genes, primers were designed from predicted gene sequences from the *L*. *salmonis* genome (http://metazoa.ensembl.org/Lepeophtheirus_salmonis, Licebase.org) and 5´ and 3´RACE were performed (see Table 1 for list of primers). We obtained full sequences of *L*. *salmonis neverland* (*Lsnvd*; Accession number (Ac. nr.)MF598470), *disembodied* (*Lsdib*; Ac. nr. ACO13011.1) and *shade* (*Lsshd*; Ac. nr. MF598469). The analysis of the genes revealed an open reading frame (ORF) of *Lsnvd* 1417 bp enconding a protein of 399 aa, *Lsdib* 1510 bp and 470 aa, and *Lsshd* 1590 bp and 530 aa. Alignment with amino acid sequences from other species (listed in Table 2) obtained through Blast search (NCBI) revealed that *Ls*Nvd contain the characteristic Rieske [2Fe-2S] and the non-heme Fe (II) domains (Fig 1A). Five conserved cytochrome P450 domains; a P/G rich motif, Helix-C, Helix-I, Helix-K, PERF and a Heme-binding motif were identified in the obtained sequences for *Ls*Dib and *Ls*Shd (Fig 1B and 1C).

### Ontogenetic analysis of *Lsnvd*, *Lsdib* and *Lsshd* using RT-qPCR

**Fig 2:**
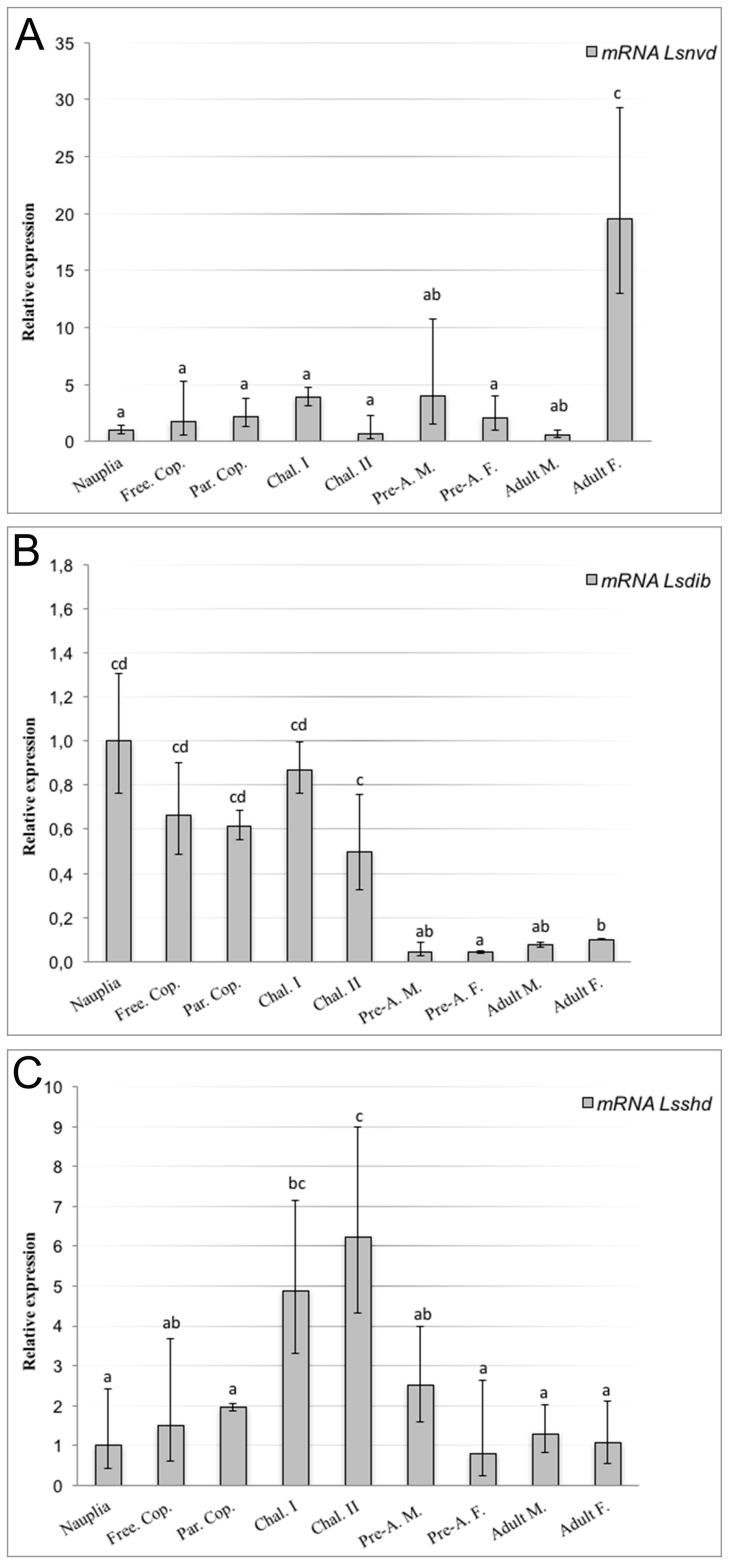
Quantitative real-time PCR analysis of relative expression in different developmental stages. A) *Lsnvd*, B) *Lsdib* and C) *Lsshd*. Each point represents the mean and confidence intervals n = 5 parallels of approx. 150 animals for the nauplius I/II and free-living copepodid (Free. Cop.) stages, n = 3 x 10 animals for the parasitic copepodid (Par. Cop.), n = 5 x 10 for the Chalimus I (Chal. I) and Chalimus II (Chal. II.) and one animal for the pre-adult male (Pre-A. M.), pre-adult female (Pre-A. F.), adult male (Adult M.) and adult female (Adult F.). The relative expression of the nauplius stage was set to 1. Different letters denote significant differences between stages (ANOVA; p < 0.05). Note the different scaling of the axes.

Expression levels for the different life stages of *Lsnvd*, *Lsdib* and *Lsshd* were obtained using RT-qPCR (see Fig 2A, 2B and 2C, respectively). The relative expression of *Lsnvd* was around five fold higher in adult females compared to the other life stages where transcript was low (Fig 2A). A significantly higher expression of *Lsnvd* was also detected in Chal I and pre-adult males compared to the adult males (Fig 2A; ANOVA, P < 0.05). In contrast to *Lsnvd*, transcript expression of both *Lsdib* and *Lsshd* was significantly higher in the early life stages of the life cycle compared to the pre-adult and adult stages (Fig 2B and 2C; ANOVA, P < 0.05). The relative expression of *Lsnvd* was more than 18 fold higher than *Lsdib* and *Lsshd* in the adult female stage, but only between 4 and 1.5 fold higher in the larval stages, respectively.

### Localisation of transcripts

**Fig 3:**
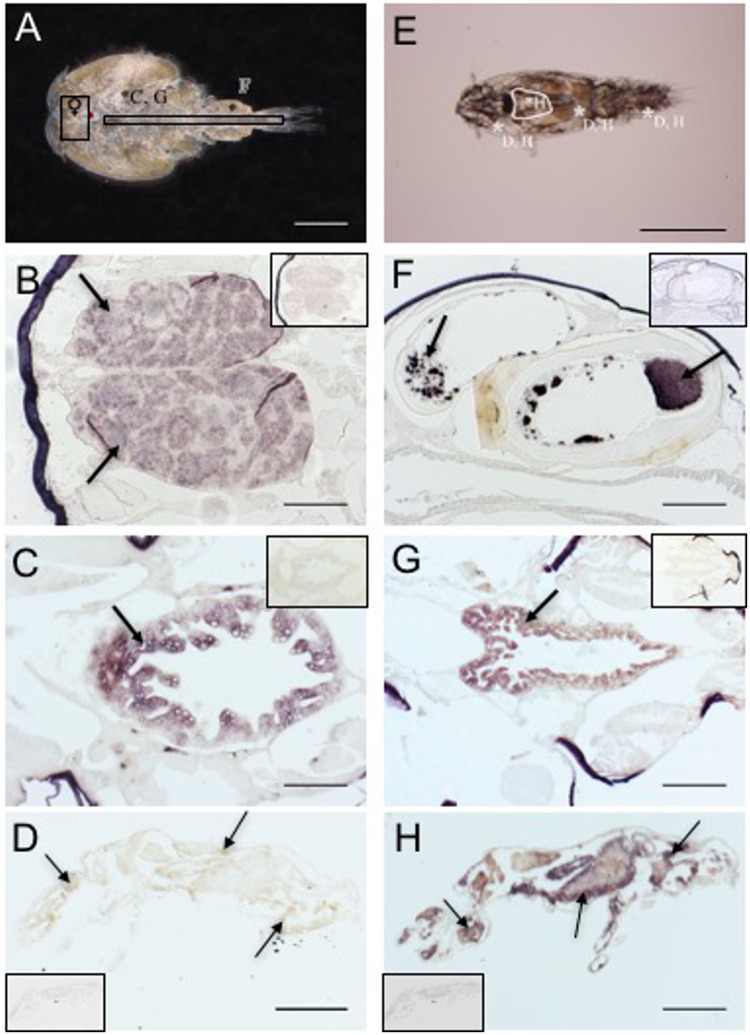
Localisation of *Lepeophtheirus salmonis Lsnvd*, B) *Lsdib* C-D) and *Lsshd* F-H) transcripts. Light microscope image of adult male louse (A) and copepodid (E) stage. Letters and asterisks are guides to the corresponding photo of individual tissues. Note that location of adult female ovaries is marked with a frame ♀in the adult male louse (A). *In situ* hybridisation was performed for each gene on sections from life stages where transcript was highest expressed using specific anti-sense RNA. Negative controls (sense RNA) applied to parallel sections are shown framed in corners. Positive staining was seen for *Lsnvd* in adult female ovaries (marked with arrows) (B). *Lsdib* was detected in the intestine of the adult female (marked with arrow) and weakly throughout the copepodid (marked with arrows) (C, D). A strong positive signal was seen for *Lsshd* in specific cells in the adult male spermatophore (marked with arrow), adult female intestine (marked with arrow) and throughout the copepodid (marked with arrows) (F, G, H). Scalebar; A) = 1000 μm; B = 300 μm; C, F, G) = 200 μm; E = 50 μm and D, H) = 100 μm.

*In situ* hybridisation was performed in order to localise the transcripts of *Lsnvd*, *Lsdib* and *Lsshd* (Fig 3B–3H). Sections of *L*. *salmonis* adult male and female louse and copepodids (7 days post molting) were selected based on the results from the expression analysis (*section 3*.*2*). Parallel sections were incubated with sense probe as a negative control. The low transcript levels of the genes made the localisation studies challenging and RNA-seq data from Licebase.org was used as support. Transcript of *Lsnvd* was detected in the ovaries of the adult female (Fig 3B) only. A weak staining in the ovaries was also observed for *Lsshd* (S2 Fig) but the strongest signal was observed in the intestine (Fig 3G). A strong signal was detected for *Lsshd* in specific but unidentified cells in the male spermatophore (Fig 3F) and in most tissues of the copepodid stage including neuronal tissue (Fig 3H). *Lsdib* transcript was weakly detected throughout the copepodid tissues (Fig 3D) and more prominently in the intestine of the adult stages (Fig 3C). Morphological assessment are based on descriptions of the reproductive anatomy described by Ritchie et al., [4].

### The ecdysteroid titer fluctuates during egg maturation

**Fig 4:**
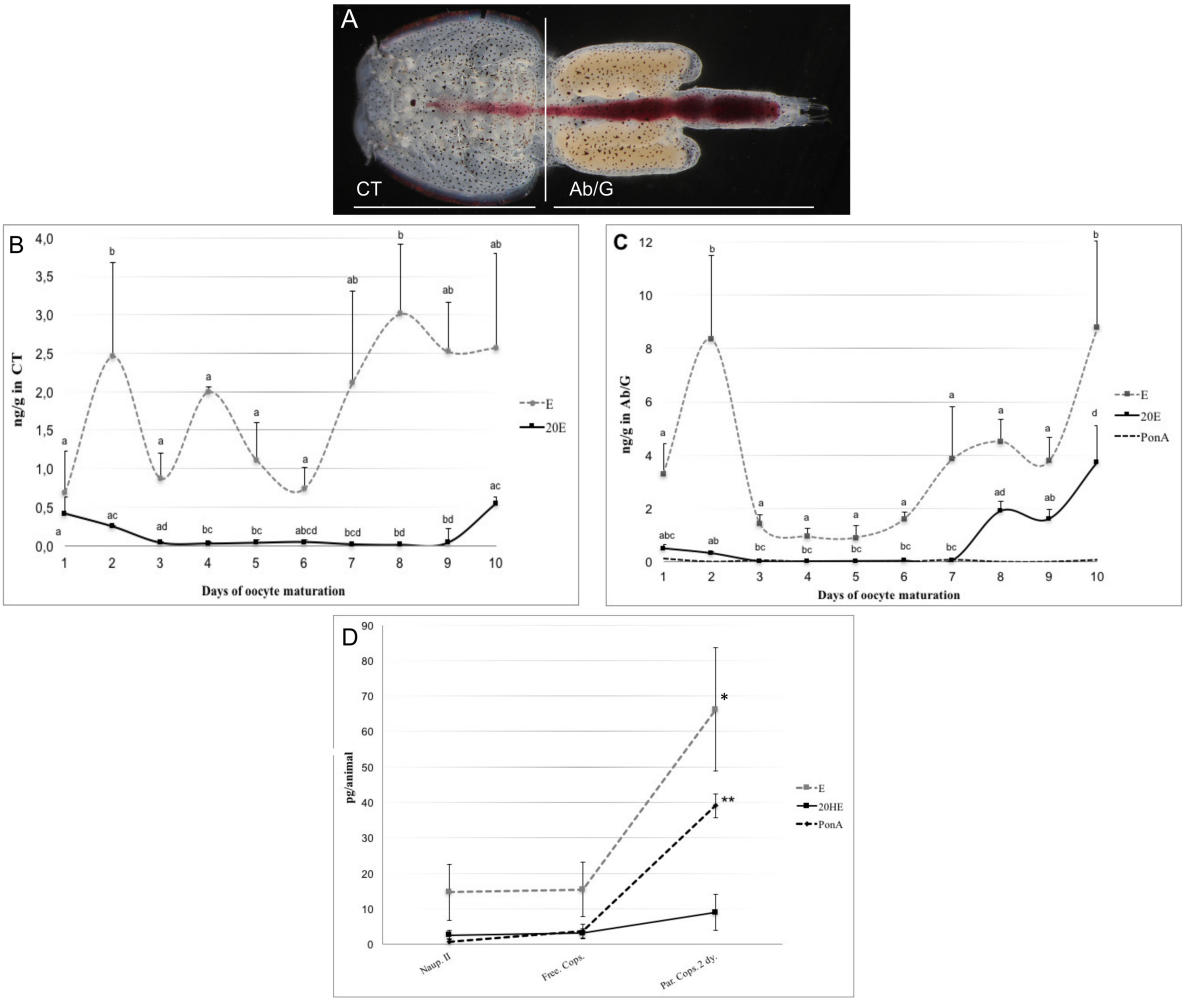
Detection of ecdysteroid level using tandem mass spectrometry. (A) Schematic presentation of separation carried out in adult female lice; CT: cephalothorax and Ab/G: abdomen/genital segment. The level of ecdysone (E), 20-hydroxyecdysone (20HE) and ponasterone A (PonA) was investigated in the cephalothorax (CT) (B), abdomen/genital segment (Ab/G) (C) and free-swimming and parasitic larval stages (D). Different letters denote significant differences between stages in B, C (ANOVA; p < 0.05). In the larval stages (D), significant difference in ecdysteroids E and PonA between stages is denoted with asterisk * and **, respectively. No difference was seen in 20HE level between larval stages. Ecdysteroid level is measured in ng/g in adult females and pg/animal in larval stages. Note different scaling of the axes and name of Y-axis.

LC/MS/MS in SRM mode (Table 3) was used for ecdysteroid analysis. The corresponding peaks in the SRM chromatograms of the reference and extracted samples were used to identify ecdysteroids present. We applied the method to identify the ecdysteroids E, 20HE and PonA in adult female lice during oocyte maturation (10 days cycle). As shown in Fig 4A, the adult female lice were divided in two parts: the cephalothorax (CT) and the abdomen/genital segment (Ab/G) containing primarily maturing oocytes in order to detect any differences in ecdysteroid content and level between the two segments. A significant difference was detected between E and 20HE concentration in both the CT (P < 0.05) and the Ab/G (P < 0.01) (Fig 4B and 4C). In addition, the total ecdysteroid content in the Ab/G segment was significantly higher compared to the CT (P < 0.05). In the CT, E concentration peaked at day two at 2.56 ng/g before a significantly drop to 0.87 ng/g was detected at day three (P < 0.05) (Fig 4B and 4C). The E concentration remained stable between days 3–6 until it increased and peaked at the end of oocyte maturation (Fig 4B, day 8, 3.01 ng/g). The same trend was observed for 20HE where 20HE level peaked at 0.54 ng/g (Fig 4B, day 10) just before the oocytes were excreted from the female. A similar pattern in the ecdysteroid titer was observed in the Ab/G segment where the E concentration dropped from 8.35 to 1.43 ng/g (P < 0.01) between days two and three before it increased from 3.78 ng/g at day nine and peaked at 8.78 ng/g day ten (Fig 4C, P < 0.05). In addition, PonA was observed at low levels around the detection limit in the extract of the Ab/G but not in the CT. PonA concentration remained low (0.061–0.131 ng/g) through oocyte maturation and no significant difference in PonA levels could be detected (Fig 4C).

### Ponasterone A is the predominant active ecdysteroid in the parasitic copepodid stage of *L*. *salmonis*

LC/MS/MS analysis was performed under the same conditions as described in *section 2*.*10* (Table 3) to investigate the ecdysteroid content and levels during larval stages. E, 20HE and PonA were all present in nauplius II and free-swimming and parasitic copepodids. No significant increase in 20HE level was detected between the larval stages, however both E and PonA increased significantly from the free-living to the parasitic copepodid stage two days after infection (Fig 4D, marked with * and ** for E and PonA, respectively; ANOVA P < 0.05). PonA was detected as the main biologically active hormone in the parasitic copepodid and peaked at ~39 (± 3,4) pg/animal compared to 20HE peaking at ~8,9 (± 5,0) pg/animal (2 d.p.i.). The E level peaked at ~66 (± 17,5) pg/animal in the parasitic copepodid stage (Fig 4D).

## Discussion

The steroid hormone 20-hydroxyecdysone is known to coordinate the execution of embryonic and post-embryonic development in many arthropods. Through a series of enzymatic steps, cholesterol is converted to the biologically active 20HE that binds to the EcR/RXR nuclear complex and initiates a range of physiological processes. The structure of the enzymes involved in ecdysteroid biosynthesis are highly conserved between insects and crustaceans, however, the physiological function in marine invertebrates has received little attention. In this study, we identified orthologs of the insect *neverland* (*nvd*) and the two cytochrome P450 enzymes *disembodied* (*dib*) and *shade* (*shd*) in *L*. *salmonis*. The three genes were selected as they represent the beginning, middle and last stage of the ecdysteroid synthesis pathway. Sequence alignment showed that the amino acid sequence of *Ls*Nvd contained a typical Rieske [2Fe-2S] binding motif and a C-terminal non-heme iron-binding domain (Fig 1A) while the conserved P450 motifs (Helix-C, Helix-I, Helix-K, a PERF-motif and a heme-binding domain) were identified in the primary structure of *Ls*Dib and *Ls*Shd (Fig 1B and 1C). This indicates that the three genes identified in *L*. *salmonis* are functionally equivalent to their insect orthologs and are part of the ecdysteroid synthesis in the salmon louse. Although the biosynthesis of ecdysteroids has not been studied in details in crustaceans these results also support the view that the biosynthetic pathway in *L*. *salmonis* is similar to the pathway present in insects.

*In situ* hybridisation was performed to identify the tissues where the ecdysteroidogenic genes are transcribed. In adult females, *Lsnvd* was found in the ovaries using *in situ* hybridisation, however, tissue specific RNA-seq data showed additional expression of *Lsnvd* transcript in the unfertilised oocytes, intestine and male testes (www.Licebase.org). These results are similar to the expression pattern found for the two *nvd* paralogs identified in *Daphnia magna (Dmnvd)* [19]. Both *Lsdib* and *Lsshd* transcript were detected in intestine and reproductive tissue of adult lice (Fig 3B, 3D and 3E) and in most tissue of the copepodid larvae (Fig 3C and 3F). In crustaceans, the activity of Halloween gene products are typically detected in ecdysone biosynthetic tissue such as the Y-organ and ovaries [10] with the exception of *shd*, which is found in a variety of peripheral tissue such as the fat body, malphigian tubules and midgut [19, 44]. In insects, ecdysteroid synthesis takes place in the prothoracic glands of larval stages and the ovaries of the adults [28, 44, 45]. The presence of both CYP genes *Lsdib* and *Lsshd* in reproductive tissue and intestine of *L*. *salmonis* suggests that both the biosynthesis of ecdysone and its conversion to the active metabolite 20HE primarily takes place in the gonads and intestine before likely being excreted into the hemolymph and distributed to target tissues. This is supported by microarray analysis of the *L*. *salmonis* intestine where all 28 cytochrome P450 genes identified in the *L*. *salmonis* genome were expressed [34]. The widespread transcript distribution of the Halloween genes in copepodids indicates that ecdysteroidogenesis is not restricted to specific tissues like the PG in immature insects.

Having established the existence of a biosynthetic pathway for production of ecdysones, further work was performed to measure the ecdysteroid level in *L*. *salmonis*. We first wanted to investigate the ecdysteroid titer during larval stages. Our studies indicate that PonA is the main biologically active hormone present in the parasitic copepodid stage, which is in accordance with prior studies performed in crustaceans [7]. After attachment of the copepodid to the host, it takes approximately five days at 10°C to molt into the next stage of the life cycle. The significant increase in E and PonA from the free living to the parasitic stage further suggests that ecdysteroids play a key role in the regulation of molting in *L*. *salmonis* as is well known from other arthropods. However, further studies of the ecdysteroid titer throughout the molting cycle is necessary in order to verify these results. Given the expression pattern and the role of ecdysone in reproduction demonstrated in other animals we wanted to explore the ecdysteroid titer during oocyte maturation. Here, measurements of the ecdysteroids clearly demonstrated the existence of E, 20HE and PonA in *L*. *salmonis*. Our study establishes that the ecdysteroid levels significantly change during maturation of the oocytes both in the CT and the Ab/G segment of female lice. The level of ecdysteroids is, however, significantly higher in the Ab/G segment compared to the CT. In addition, PonA was present in the Ab/G segment at very low levels but below the detection level in the CT, which implies that this steroid is utilized by the vitellogenic oocytes but not by the ovaries in the adult female. A drop in E and 20HE level is evident at the start of vitellogenesis before both ecdysteroids increase in concentration at the end of oocyte maturation. A similar increase in ecdysteroids in the oocytes is seen in the shore crab *Carcinus maenas* [46] where ecdysteroids are necessary for initiation of oocyte maturation. Higher concentrations of ecdysteroid levels were also observed in female *Calanus finmarchicus* with mature egg sacs indicating that ecdysteroids are involved in egg maturation and reproduction [47]. RNA-seq data from *L*. *salmonis* (www.Licebase.org) shows that several members of the CYP450 genes involved in ecdysteroid synthesis are expressed in the unfertilized eggs. This suggests that the oocytes are capable of *de novo* synthesis of ecdysteroids in *L*. *salmonis*.

In summary, we identified the genes *neverland*, *disembodied* and *shade* involved in the biosynthesis of ecdysteroids, in the salmon louse, *L*. *salmonis*. Transcript expression of the ecdysteroid biosynthetic genes in the reproductive tissue and the increase in ecdysteroid level during oogenesis strongly indicates that ecdysteroids play a key role in oocyte maturation in salmon lice. In addition, measurements of ecdysteroids during larval stages implies that PonA is the biologically active ecdysteroid involved in molting during larval stages. However, functional studies are essential in order to determine the importance of the biosynthetic ecdysteroid genes in the salmon louse life cycle.

## Supporting information

S1 Fig Quantitative real-time PCR analysis of *Lsnvd* (A), *Lsdib* (B) and *Lsshd* (C) transcript expression in different life stages of the salmon louse. The graph shows the measured mRNA level from each biological sample during ontogenesis. Note that the graphs (A-C) shows the dCT values used to calculate the relative expression of each gene given in Fig 2.
(TIF)

S2 Fig Localisation of *Lsshd* transcript using *in situ* hybridization in adult female ovaries. Close-up of ovaries. Scalebar = 50 μm.
(TIF)

S3 Fig Detection of ecdysteroid level using tandem mass spectrometry in adult female lice. Represented in the graphs are the levels of the ecdysteroids E (A, C), 20HE (B, D) and PonA (E) measured in each individual biological sample (n = 5) of the cephalothorax (CT; A,B) and the abdomen/genital segment (Ab/G; C-E) of adult female lice.
(TIF)

S4 Fig Detection of ecdysteroid level using tandem mass spectrometry during larval stages. Represented in the graphs are the levels of the ecdysteroids E (A), 20HE (B) and PonA (C) measured in each individual biological sample (n = 3) of nauplius, free-living copepodids and the parasitic copepodid (2 days post infection) life stages of the salmon louse.
(TIF)

## Abbreviations

LC/MS/MS: Liquid chromatography tandem mass spectrometry
RT-qPCR: real-time quantitative PCR
