# Supplementary figures and images for: Identification and characterisation of the ecdysone biosynthetic genes neverland, disembodied and shade in the salmon louse Lepeophtheirus salmonis (Copepoda, Caligidae)
Source: PLoS One. 2018 Feb 5;13(2):e0191995. doi: 10.1371/journal.pone.0191995 (PMC5798790; doi:10.1371/journal.pone.0191995)

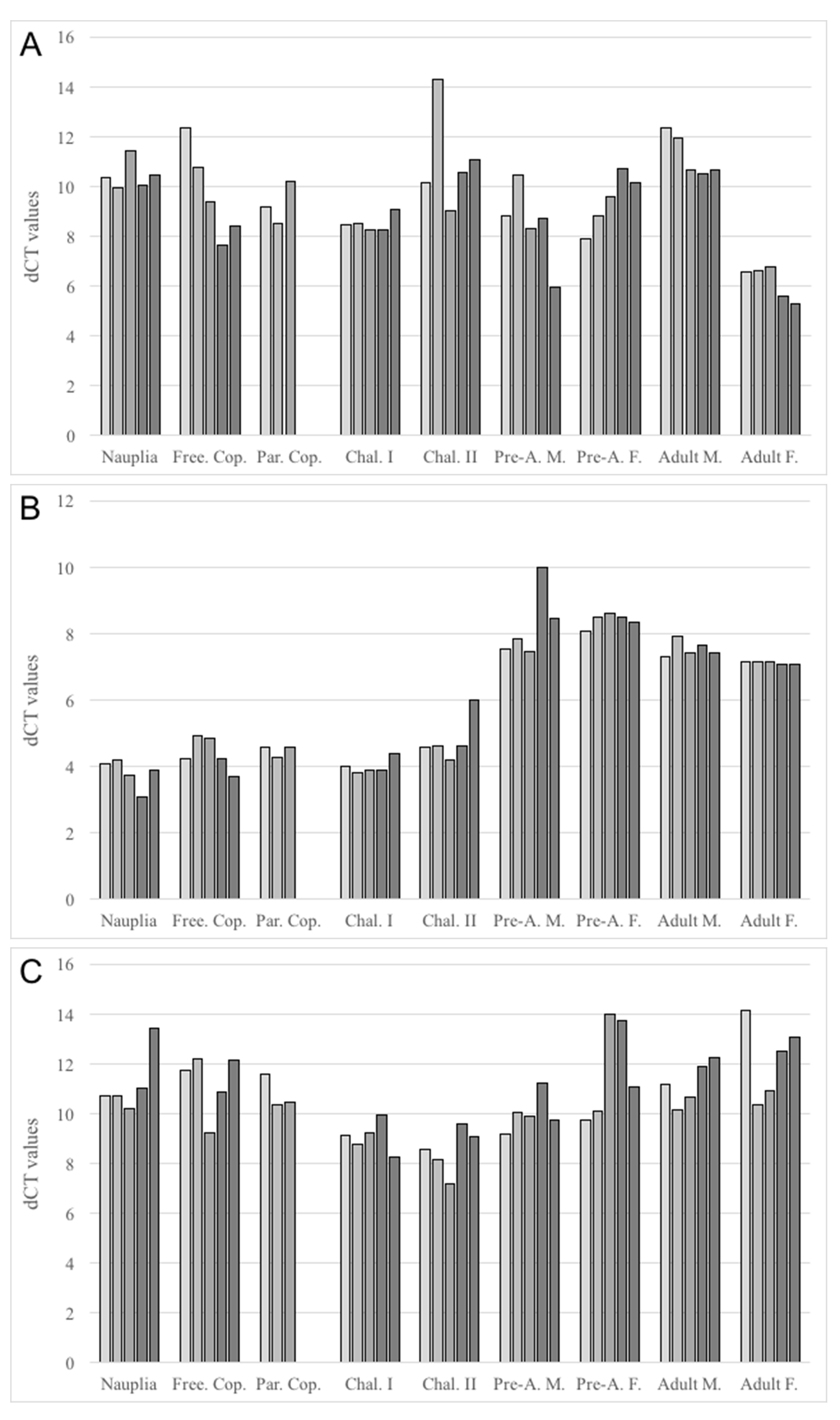

Supplement: S1 Fig — The graph shows the measured mRNA level from each biological sample during ontogenesis. Note that the graphs (A-C) shows the dCT values used to calculate the relative expression of each gene given in Fig 2. (TIF) [file pone.0191995.s001.tif]

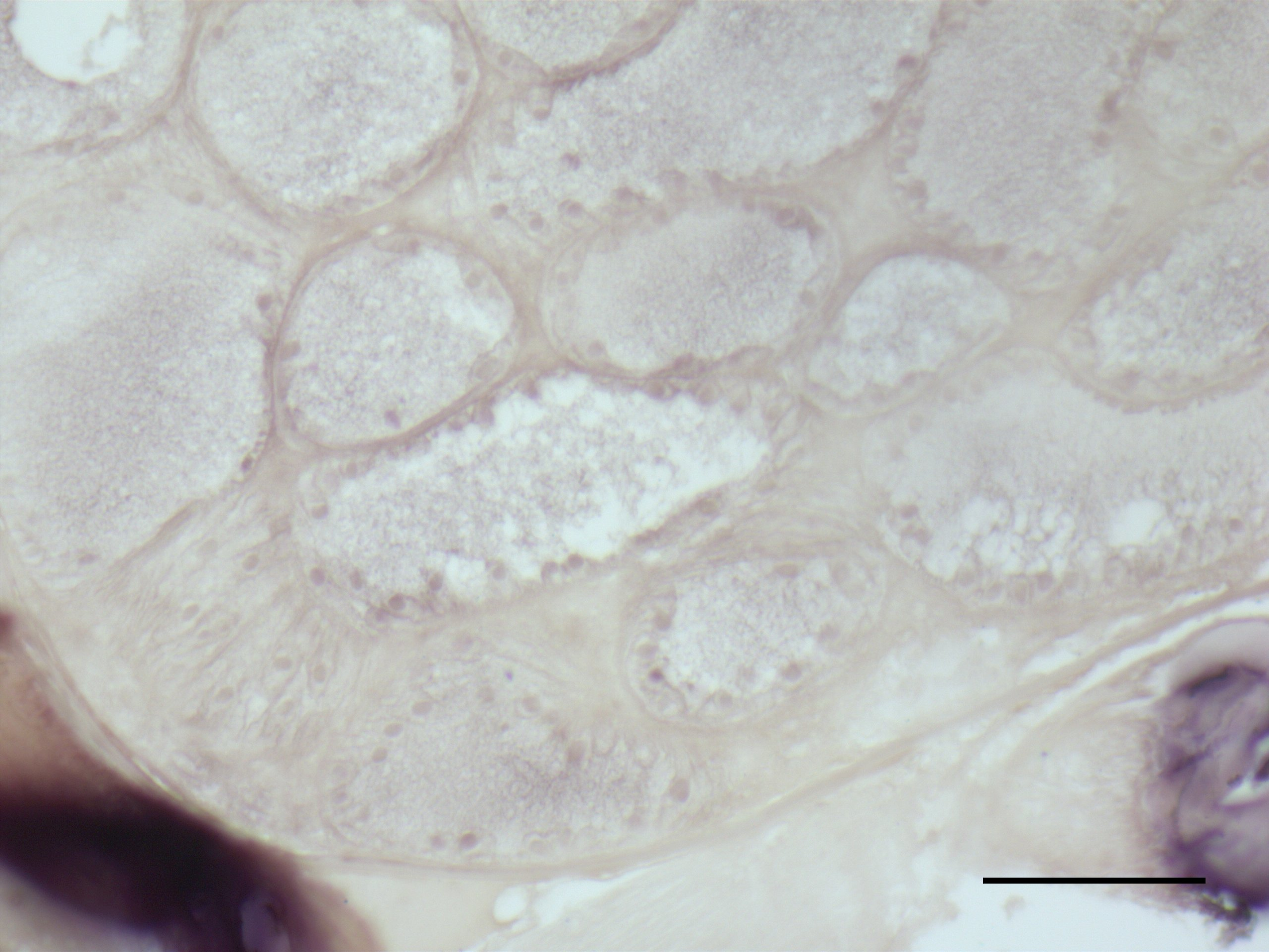

Supplement: S2 Fig — Close-up of ovaries. Scalebar = 50 μm. (TIF) [file pone.0191995.s002.tif]

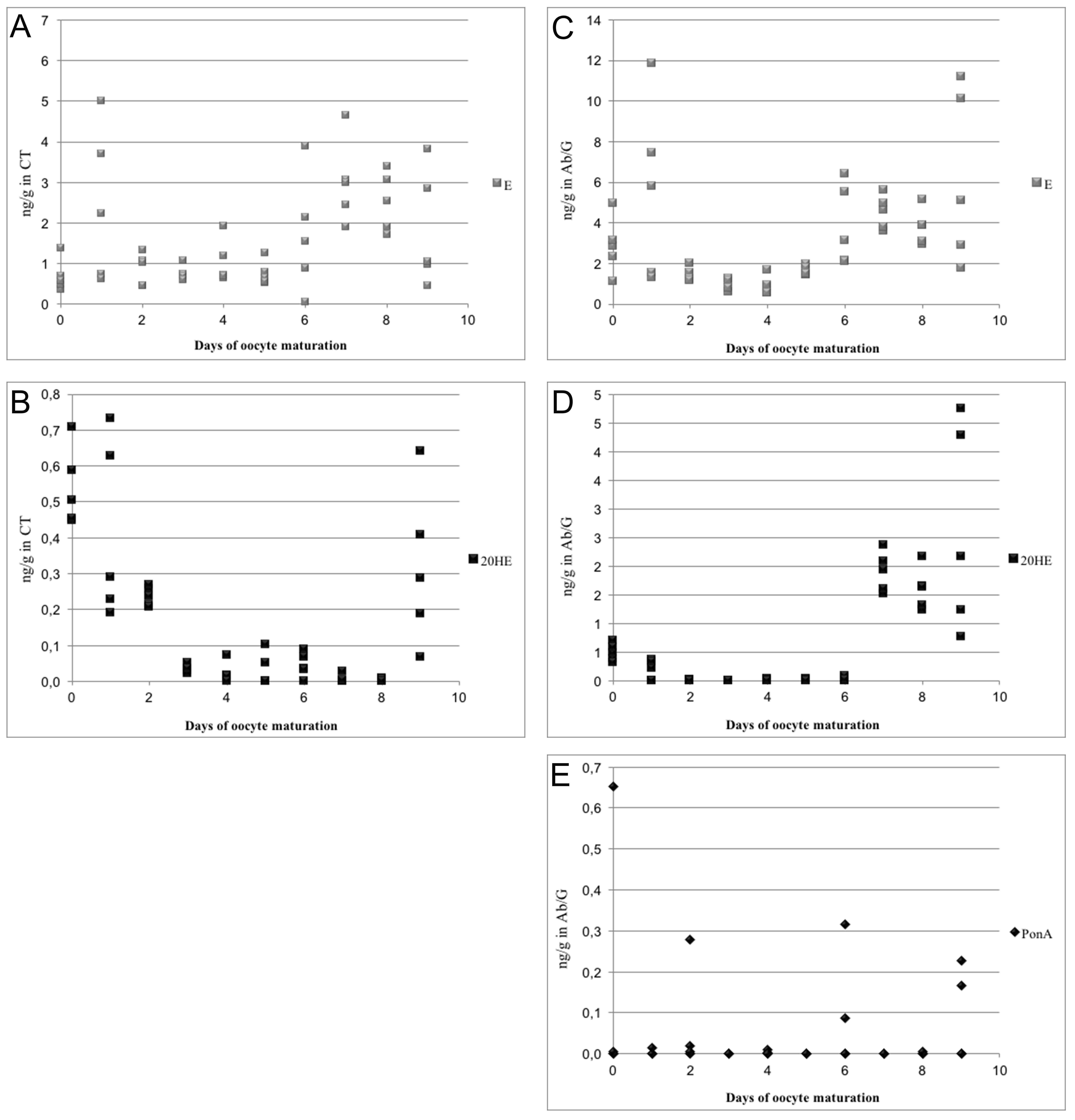

Supplement: S3 Fig — Represented in the graphs are the levels of the ecdysteroids E (A, C), 20HE (B, D) and PonA (E) measured in each individual biological sample (n = 5) of the cephalothorax (CT; A,B) and the abdomen/genital segment (Ab/G; C-E) of adult female lice. (TIF) [file pone.0191995.s003.tif]

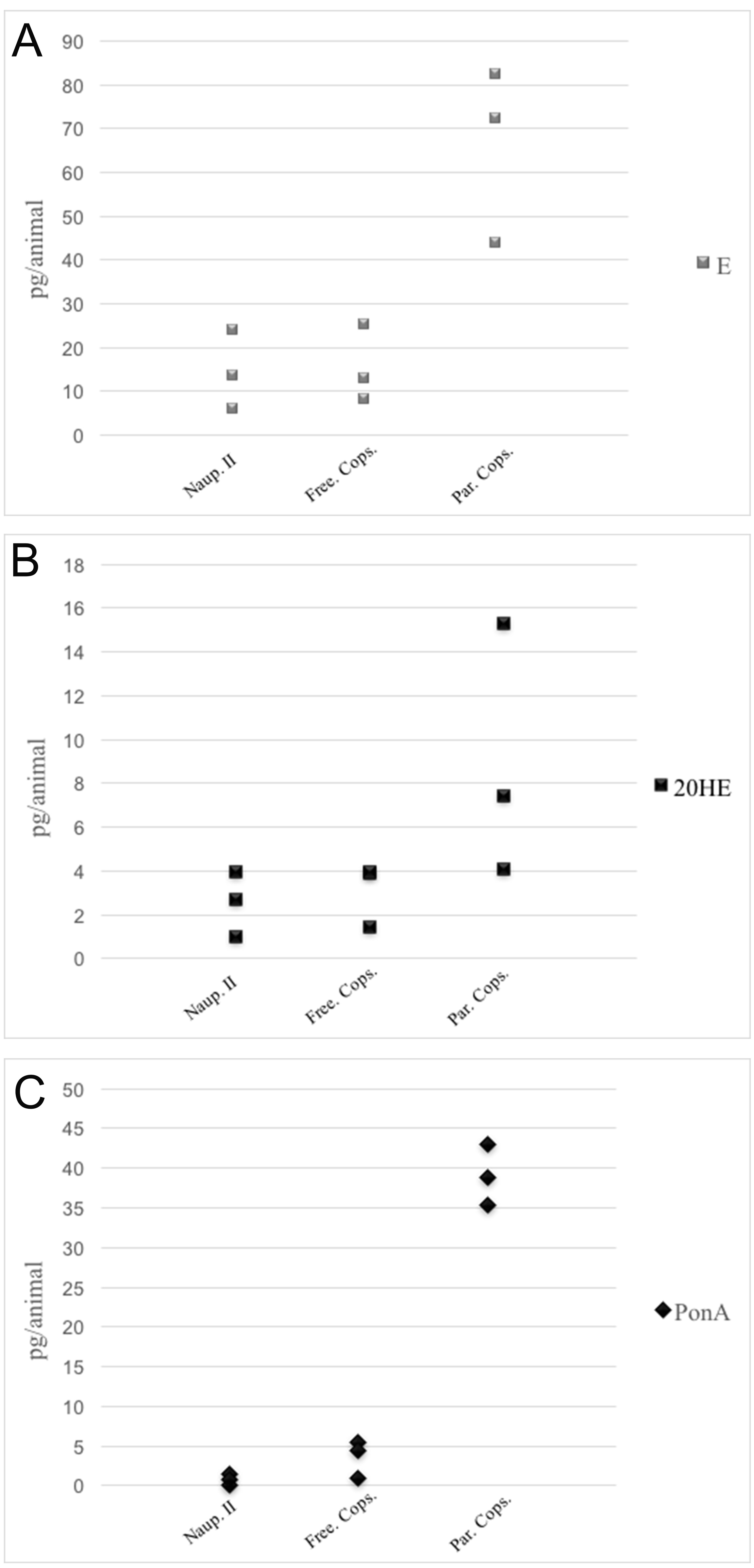

Supplement: S4 Fig — Represented in the graphs are the levels of the ecdysteroids E (A), 20HE (B) and PonA (C) measured in each individual biological sample (n = 3) of nauplius, free-living copepodids and the parasitic copepodid (2 days post infection) life stages of the salmon louse. (TIF) [file pone.0191995.s004.tif]
